# Editorial: Affective, cognitive and social neuroscience: lifelong knowledge and challenges in the post-pandemic world and virtual dimension, volume II

**DOI:** 10.3389/fpsyg.2025.1596292

**Published:** 2025-04-10

**Authors:** Rosalba Morese, Sara Palermo

**Affiliations:** ^1^Faculty of Communication, Culture and Society, Università della Svizzera Italiana, Lugano, Switzerland; ^2^Department of Psychology, University of Turin, Turin, Italy; ^3^Neuroscience Institute of Turin (NIT), University of Turin, Turin, Italy

**Keywords:** post-pandemic behavior, virtual environments, consumer behavior, social cognition, emotional regulation

This Research Topic explores the profound and complex ways in which the COVID-19 pandemic, along with the spread of virtual environments, has affected human behavior and mental processes. This Research Topic includes an in-depth exploration of the latest findings in cognitive science, affective neuroscience and social neuroscience, focusing on neuropsychological processes and brain circuits that regulate fundamental aspects of human behavior. The pandemic has exposed our vulnerability and disrupted global socio-economic systems and personal lives. This emphasizes the need to rethink outdated knowledge frameworks and find innovative solutions to new and emerging societal needs.

In particular, prolonged COVID has been associated with significant neurological sequelae, cognitive dysfunction and behavioral changes that affect daily life and autonomy (Celeghin et al., 2024). These effects are not only due to direct neuroinvasion and multi-organ dysfunction but are also related to mental health problems and broader socioeconomic changes. Understanding these mechanisms is critical to developing patient-centered approaches that address both cognitive and emotional challenges.

At the same time, the pandemic has exacerbated feelings of loneliness and social isolation, particularly among older people. Research suggests that these experiences are influenced by attachment styles and deficits in social cognition, which can accelerate neurodegeneration and negatively impact overall wellbeing (Morese and Palermo, 2022). Social neuroscience emphasizes the importance of meaningful affective relationships in mitigating the negative effects of isolation and preventing cognitive decline.

This Research Topic builds on two previous editorials in the series: *Editorial: Affective, cognitive and social neuroscience: new knowledge in normal aging, minor and major neurocognitive disorders* (Morese et al., 2022) and *Editorial: Perspective-taking, self-awareness and social cognition in neurodegenerative disorders, cerebral abnormalities and acquired brain injuries (ABI): A Neurocognitive Approach* (Palermo et al., 2020). Previous works have examined how neural mechanisms are related to aging, neurocognitive disorders, and social cognition in the context of neurodegenerative disorders. In this third volume, the focus shifts to how the pandemic and virtual environments have reshaped emotional regulation, social interaction, and cognitive functioning. These developments present new challenges but also unique opportunities for understanding the human brain in an increasingly complex and digitally connected world.

The Research Topic contains five insightful articles that explore different facets of affective, cognitive and social neuroscience in the post-pandemic era.

Li and Zheng examine the influence of the language style of anchors in advertisements and product promotions on consumers' purchase intentions. Their study highlights the impact of formal and informal language on consumer perceptions and decision-making. The authors find that informal language creates a stronger personal connection with consumers and therefore increases the likelihood of purchase behavior. In contrast, formal language is perceived as authoritative but less personable. This article offers valuable insights into optimizing marketing communications to improve customer loyalty and purchase behavior.

Liang et al. examine the impact of mask-wearing on consumer behavior, particularly in relation to the desire for uniqueness when making purchase decisions. The authors argue that by both concealing identity and acting as a form of personal expression, masks can encourage consumers to seek out products that emphasize individuality. The study shows that wearing a mask increases the likelihood of uniqueness-seeking behavior, as the psychological distance created by the mask allows individuals to make bolder and more distinctive choices. This study offers important insights into how societal changes, such as the wearing of masks, can influence consumer psychology and behavior.

Saito et al. examine how perceptions of humanity differ across age groups and explore the neural and behavioral processes underlying these perceptions. The study examines how the brain processes faces and emotional expressions of people of different ages—children, adults and the elderly. Using neuroimaging, the study identifies areas of the brain that are activated during these perceptions and shows that while the recognition of humanity remains constant, emotional and behavioral responses vary depending on the age of the person being observed. This study provides valuable insights into how our social brains process human-related cues and has implications for improving intergenerational interactions.

Lv et al. explore the “gaze effect” and examine how the presence or suggestion of gaze influences prosocial behavior and satisfaction in economic decision-making. Using the dictator game, the study shows that participants are more likely to make generous decisions when they are reminded—even indirectly—that they are being watched. The results suggest that awareness of being watched influences both prosocial behavior and personal satisfaction, as individuals tend to allocate more resources to others when exposed to images or symbols that represent eyes. The article emphasizes how the act of giving is shaped not only by external social pressures but also by internal feelings of moral satisfaction and social conformity.

Xie et al. examine the neural basis of value hierarchies, focusing on how emerging adults prioritize and process personal values in decision-making. The study introduces a typology of value profiles and examines how individuals prioritize social, economic, and personal goals. Using neuroimaging, the authors identify brain regions that are activated when participants make decisions based on these values, revealing distinct neural patterns in young adults. The results suggest that value-based behavior is highly individualized and evolves during this critical phase of life. This research provides valuable insights into the neural mechanisms underlying value-based decision-making in young adulthood.

Taken together, these five articles shed light on the intricate relationships between cognitive, emotional and social processes in the post-pandemic world and virtual environments. They make an important contribution to understanding how neuroscience can inform our approach to human behavior in rapidly changing contexts and offer new perspectives on emotional regulation, decision-making and social dynamics in this new era.
